# Synthesis and Characterization of Cellulose Triacetate Obtained from Date Palm (*Phoenix dactylifera* L.) Trunk Mesh-Derived Cellulose

**DOI:** 10.3390/molecules27041434

**Published:** 2022-02-21

**Authors:** Hamid M. Shaikh, Arfat Anis, Anesh Manjaly Poulose, Saeed M. Al-Zahrani, Niyaz Ahamad Madhar, Abdullah Alhamidi, Saleh Husam Aldeligan, Faisal S. Alsubaie

**Affiliations:** 1 SABIC Polymer Research Centre, Department of Chemical Engineering, King Saud University, P.O. Box 800, Riyadh 11421, Saudi Arabia; aarfat@ksu.edu.sa (A.A.); apoulose@ksu.edu.sa (A.M.P.); szahrani@ksu.edu.sa (S.M.A.-Z.); akfhk90@hotmail.com (A.A.); 442100416@student.ksu.edu.sa (S.H.A.); 437102504@student.ksu.edu.sa (F.S.A.); 2 Department of Physics and Astronomy, College of Sciences, King Saud University, Riyadh 11451, Saudi Arabia; nmadhar@ksu.edu.sa

**Keywords:** cellulose, cellulose triacetate, date palm mesh, acetylation, degree of substitution, thermal stability

## Abstract

Cellulosic polysaccharides have increasingly been recognized as a viable substitute for the depleting petro-based feedstock due to numerous modification options for obtaining a plethora of bio-based materials. In this study, cellulose triacetate was synthesized from pure cellulose obtained from the waste lignocellulosic part of date palm (*Phoenix dactylifera* L.). To achieve a degree of substitution (DS) of the hydroxyl group of 2.9, a heterogeneous acetylation reaction was carried out with acetic anhydride as an acetyl donor. The obtained cellulose ester was compared with a commercially available derivative and characterized using various analytical methods. This cellulose triacetate contains approximately 43.9% acetyl and has a molecular weight of 205,102 g·mol^−^^1^. The maximum thermal decomposition temperature of acetate was found to be 380 °C, similar to that of a reference sample. Thus, the synthesized ester derivate can be suitable for fabricating biodegradable and “all cellulose” biocomposite systems.

## 1. Introduction

Developing sustainable products from biobased polysaccharides has become the primary focus of research in academia and industry for various applications. Moreover, increasing lignocellulosic biomass transformation into valuable polymers is viable for addressing major worldwide environmental concerns caused by synthetic plastic pollution. In particular, the applications of different forms of cellulose and its derivatives have received increasing attention due to their desirable properties, such as biodegradability, biocompatibility and sustainability. Cellulose, an environmentally innocuous polysaccharide, is the most abundant resource available to humans, having a natural production capacity of 10^11^–10^12^ tons every year [1]. Cellulose can be utilized by obtaining (a) biopolymers from cellulose-derived monomers, (b) the extraction of nanocellulose, and (c) the modification for various polymeric derivatives. Among these derivatives, cellulose esters and ethers are the most common.

Cellulose acetate is an important derivative due to its wide range of applications in the fields of membranes, packaging films, optical devices and polymer composites [2,3,4,5]. On a commercial scale, cellulose acetate is produced using purified cellulose obtained primarily from wood pulp and cotton linters. However, many forest resource conservation regulations restrict the use of wood, even though market demand is constantly increasing. As a result, various additional sources of cellulose, mainly agricultural non-wood residue waste, have been continually explored to obtain a suitable grade of cellulose for further use. Cellulose acetate is prepared heterogeneously or homogeneously with activating solvents, and then, the cellulose reacts with an acetyl donor in the presence of a catalyst. All three hydroxyl groups of cellulose become acetylated in this reaction, resulting in the formation of cellulose triacetate (CTA) with a degree of substitution (DS) in the range of 2.7–3 [6]. However, subsequent partial hydrolysis of cellulose triacetate is required to obtain cellulose acetate with a lower degree of substitution [7].

In the past, some waste materials, including agricultural residue, were used to synthesize cellulose triacetate. Suzuki Shiori et al. produced cellulose acetate from sugarcane bagasse by using 1-ethyl-3-methylimidazolium acetate as a solvent as well as a catalyst and isopropenyl acetate as an acetyl donor. However, this reaction was carried out in homogenous media at 50 °C and yielded acetone-soluble cellulose acetate with a degree of substitution from 2.5–2.8 [8]. Similarly, sugarcane bagasse was used to isolate pure cellulose first and then acetylated heterogeneously to obtain cellulose triacetate with a degree of substitution of 2.6–3 [9].

Additionally, sweet sorghum bagasse (*Sorghum bicolor* (L.) Moench) was used for cellulose triacetate synthesis. The bagasse was first pretreated with hydrogen peroxide, sodium chlorite, acetic acid and sodium hydroxide to obtain pure cellulose. This cellulose was then subjected to an acetylation reaction, and cellulose triacetate was reported to be obtained [10]. Similarly, palm oil bunches and dried jackfruit leaves were utilized to obtain cellulose and then cellulose ester. In this study, 36.45% and 13.72% cellulose were obtained, and 81.75% and 63.89% cellulose ester were obtained from palm oil bunches and jackfruit leaves, respectively [11]. Moreover, agro-industrial waste such as royal palm tree leaf sheath was used to prepare cellulose triacetate. Complete conversion into cellulose acetate was achieved with a degree of substitution ranging from 2.08 to 2.82 and a yield of approximately 99.5% [12]. Similarly, biomass waste from olive oil was used to extract cellulose, followed by the synthesis of cellulose triacetate. After a series of pretreatment steps, ~35% cellulose was obtained and then treated with an N, N dimethylacetamide (DMAc)/lithium chloride solvent system with acetyl chloride as an acetyl donor. Cellulose triacetate with a degree of substitution (DS) of 2.76 was reported to be obtained [13]. In addition to these agricultural waste residues, industrial wastes such as towels [14], cotton fabrics [15], cigarette butts [16], empty tissue rolls and egg trays [17] have been explored for cellulose acetate production.

Date palm trees (*Phoenix dactylifera* L.) are one of the most farmed plants in the arid region and contain a high amount of lignocellulosic fiber. Saudi Arabia had already cultivated 30 million date palm trees in 2018 [18], and as per the green Saudi Initiative Program, 10 billion trees, predominantly palm dates, will be planted in the coming decade. Consequently, during seasonal trimming and refinement of the palms, significant quantities of waste biomass accumulate. It is estimated that each palm tree produces 20 to 35 kg of biomass and that approximately 1 million metric tons of biomass waste are accrued each year [19]. This lignocellulosic waste biomass has the potential to be a sustainable source of cellulose and its derivatives for a variety of uses.

Furthermore, only a few studies on the utilization of waste date palm lignocellulose have been performed in the past. A study has previously been published on using date palm fronds for the isolation and modification of cellulose. In this study, cellulose was derived from fronds and acetylated to obtain cellulose acetate. Cellulose acetate, which has solubility in chloroform (DS-3) and acetone (DS-2.4), was reported to be obtained [20]. Microfibrilated cellulose was extracted from date seeds in another study. Acetylation was carried out in a heterogeneous manner. Optimal reaction parameters, such as the reactant ratio, catalysis amount, time, and temperature, were investigated to obtain the highest yield of cellulose triacetate. The optimum conversation yield was reported to be ~80%, with a degree of acetyl substitution of ~3 [21].

In a previous study, we isolated pure cellulose from palm date trunk mesh fibers to make nanocellulose [18]. To further investigate the suitability of this cellulose in this work, we first performed derivatization to make the ester of this cellulose and perform detailed characterizations of the structural-property relationship. This cellulose acetate was also compared to commercially available cellulose triacetate and found to have comparable characteristics.

## 2. Results and Discussion

### 2.1. Assignment of the Chemical Functionality

**Figure 1:**
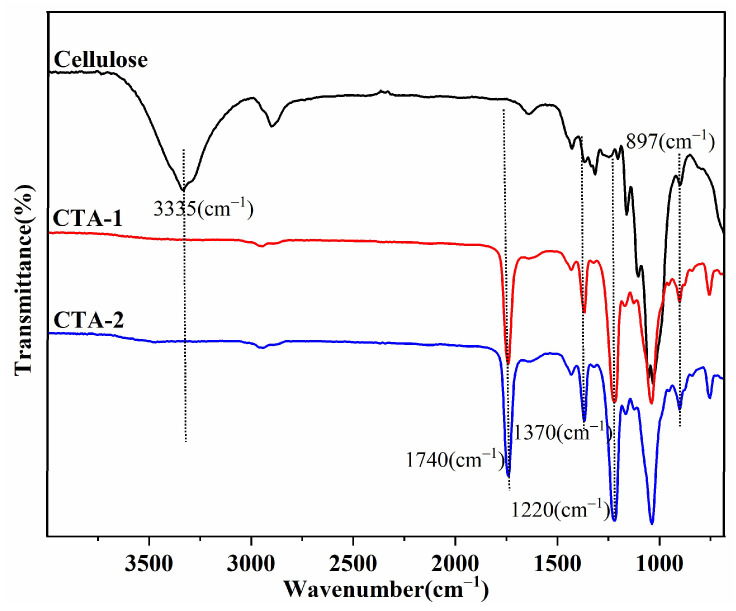
FTIR spectra of cellulose triacetate (CTA) samples.

The FTIR spectra of cellulose acetate samples are shown in Figure 1, and Table 1 details the chemical functional groups of cellulose and its ester derivative.

The strong bands at 3335 cm^−1^ in cellulose are due to –OH stretching, while 1052 cm^−1^ is a characteristic band attributed to C–O–C in anhydroglucose units of cellulose. The complete disappearance of the –OH frequency of cellulose and the appearance of the carbonyl (C=O) band at 1740 cm^−1^ in cellulose acetate confirm the successful chemical conversion to an ester. Similarly, all spectral positions for the synthesized cellulose acetate(CTA-1) versus commercial cellulose acetate(CTA-2) are also tabulated in Table 1. These FTIR spectra matched well with each other while functional groups analysis and bonding type conformations directly suggest the similar chemical structure.

Furthermore, the acetyl group’s C–H bending and C–O stretching vibrations are also observed at approximately 1370 cm^−1^ and 1220 cm^−1^, respectively. Moreover, cellulose acetate samples were found to be free from acetic acid and acetic anhydride because of the absence of bands at 1760–1840 and 1700 cm^−1^ [6]. Interestingly, the intensity of the cellulose backbone (C–O–C), which is at approximately 1052 cm^−1^, remains unaltered, revealing that it does not participate in the reaction and that only the –OH groups are acetylated. The crystalline cellulose phase is associated with the peak at approximately 1420—1430 cm^−1^, while the amorphous component of cellulose is assigned to the band at 897 cm^−1^ [22]. The intensity of the amorphous region in cellulose acetate (characteristic amorphous peak ~897 cm^−1^ in cellulose) increased compared with cellulose. The acetylation process confirmed that this reduces cellulose’s crystalline structure by destroying hydrogen bonding between the –OH groups, thereby facilitating solubility in common organic solvents. It should be mentioned that the initial cellulose is insoluble in most common organic solvents, and the degree of substitution of the acetyl groups determines the solubility of cellulose acetate in various solvents.

### 2.2. ^1^H-NMR Spectroscopic Analysis

**Figure 2:**
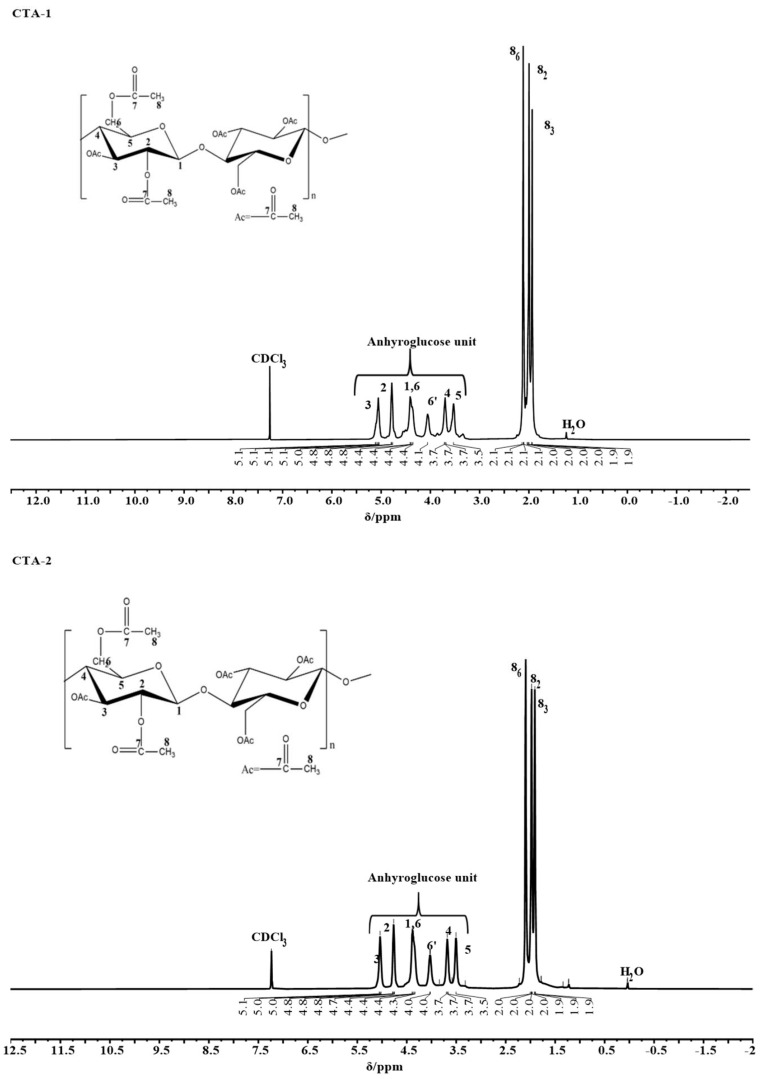
^13^C-NMR spectral characteristics of the CTA sample.

Since these cellulose acetate samples can be completely dissolved in chloroform, deuterated chloroform (CDCI_3_) was chosen as a solvent for ^1^H-NMR analysis. Figure 2 displays the ^1^H-NMR spectra of cellulose triacetate (CTA-1 and CTA-2) samples. All chemical shifts at 7.2 ppm are referenced for the solvent.

In CTA-1, the hydrogen atoms in methyl (–CH_3_) groups of acetyl groups were detected at approximately 2.1 ppm. The signal at 1.9 ppm corresponds to hydrogen connected to the third carbon atom (C3) in the cellulose skeleton, while the signal at 2.1 ppm corresponds to the hydrogen of the sixth carbon atom (C6). Similarly, in the anhydrous glucose unit, the proton signal coupled to the C1 atom was detected at 4.4 ppm, with other signals found at 4.8 ppm for C2, 5.1 ppm for C3, 3.7 ppm for C4, 3.5 ppm for C5, and 4.3 ppm and 4.0 ppm for C6 and C6′, respectively [14]. Comparably, synthesized cellulose acetate (CTA-2) also showed a similar ^1^H-NMR spectrum in which the hydrogen atoms in methyl (–CH_3_) groups of acetyl groups were detected at approximately 2.1 ppm, 2.0 ppm, and 1.9 ppm for C6, C2, and C3, respectively. The proton signal coupled to the C1 atom was detected at 4.4 ppm, with other signals found at 4.7 ppm for C2, 5.0 ppm for C3, 3.7 ppm for C4, 3.5 ppm for C5, 4.1 ppm for C6, and 4.04 ppm for C6′ of the anhydrous glucose unit.

### 2.3. ^13^C-NMR Analysis

**Figure 3:**
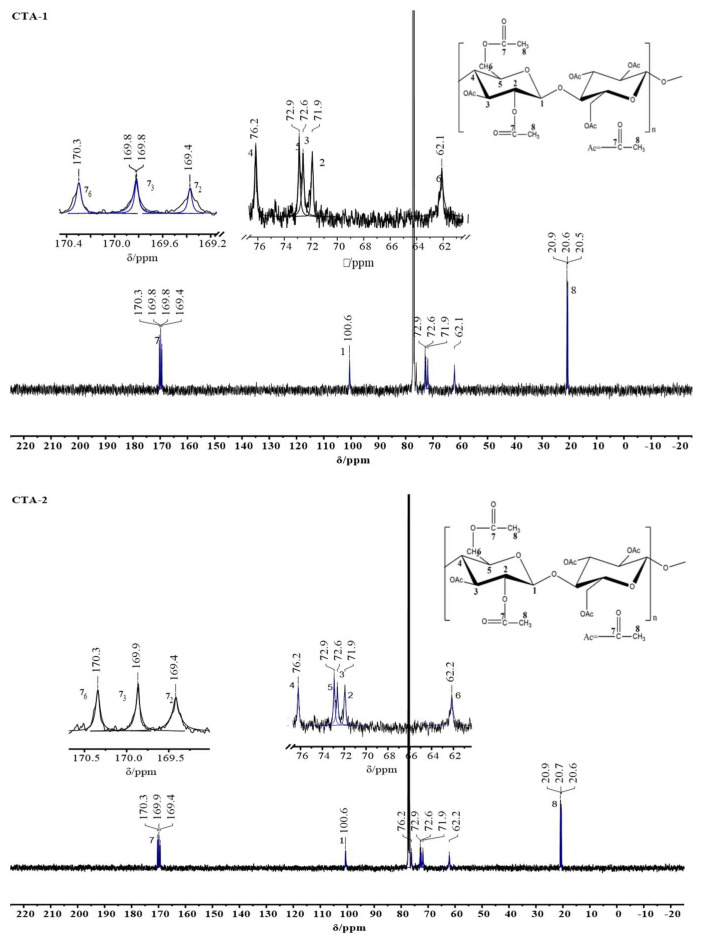
^13^C-NMR spectral characteristics of the CTA sample.

Furthermore, the ^13^C-NMR spectra of the CTA samples are given in Figure 3, and the signal positions of the specific carbons are summarized in Table 2. The carbon atoms in the carbonyl group (C7) are located between 169 and 170 ppm. The first carbon atom is located at approximately 100 ppm, whereas the second to fifth carbon atoms are distributed between 71 and 77 ppm. The sixth and eighth carbons were found at 61–62 ppm and 20–21 ppm, respectively [26].

### 2.4. Wide-Angle X-ray Diffraction (WAXRD)

**Figure 4:**
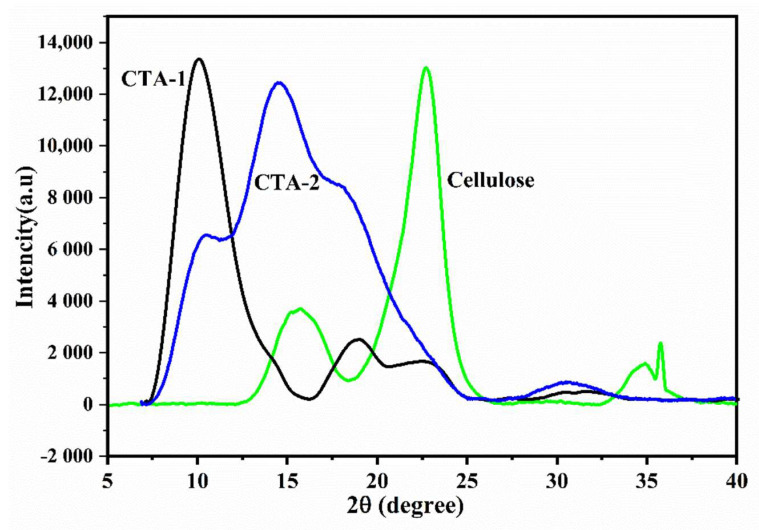
XRD diffractograms of cellulose, CTA-1 and CTA-2.

XRD diffractograms of cellulose triacetate can be used to investigate the acetylation of cellulose. It is well-known that the heterogeneous acetylation process induces semicrystalline characteristics in cellulose acetate. The acetylation process took place in stages and phases, with the cellulose surface being continually exposed to and dissolved by the reaction reagents. Then, a new cellulose surface can be obtained, and the acetylation process on cellulose begins. These reaction conditions are maintained until a homogenous environment in the solution is obtained. As shown in Figure 4, the strong diffraction peaks of the characteristic cellulose crystal planes at 2θ values of 15.7°, 22.7°, and 35.8° were altered, and a noticeable peak was observed at ~8° (2θ: 8.1 in CTA-1 and 8.5 in CTA-2). Furthermore, this peak is associated with semicrystalline acetylated cellulose derivatives.

Other weak intensity diffraction peaks were detected at 2θ values of 17.50°, 21.29° in CTA-1 and 13.01°, 16.68°, and 30.16° in CTA-2, which suggests the cellulose triacetate structure [9]. These peaks are caused by an increase in the interfibrillar distance induced by the presence of acetyl groups along with cellulose chains [27]. It is also observed that CTA-2 is more brittle than CTA-1. However, the crystallinity and brittleness of cellulose acetate can be attributed to the initial source of cellulose and the overall reaction conditions employed for the synthesis.

### 2.5. Thermal Analysis

**Figure 5:**
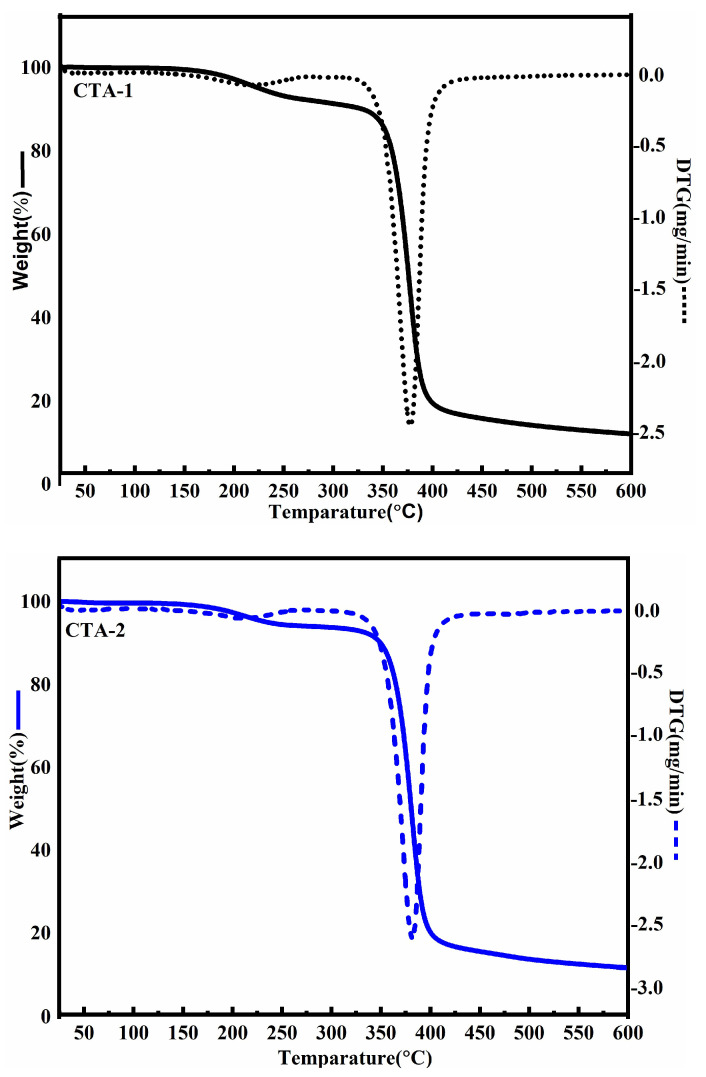
Thermogravimetric (TGA) and differential thermogravimetric (DTG) analysis of cellulose acetate samples.

Thermal analysis of cellulose acetate samples was carried out to predict the thermal stability of the samples. As shown in Figure 5, three different degradation events occur in both samples, with moisture and volatile substances being removed at temperatures ranging from 50 to 150 °C.

The weight-loss stage in the range of 150–315 °C comprised first the decomposition of the esterified chains of cellulose acetate (~190–245 °C), and subsequently, the degradation of the cellulose skeleton (~245–315 °C) occurred. Near this temperature range, cellulose undergoes dehydration, depolymerization, and the decomposition of glycosidic units, resulting in the formation of carbon residue [21,28]. The DGT curve was used to determine the maximum weight loss, which was 378 °C for CTA-1 and 382 °C for CTA-2. These findings are consistent with previous research in which CTA was shown to have the maximum weight loss between 370 °C and 380 °C [12,14,21]. Moreover, the onset and end thermal degradation temperatures of both samples were found to be in the ranges of 170–320 °C and 420–430 °C, respectively. Chen J et al. previously synthesized cellulose triacetate with a degree of substitution of 2.90 using a pure cellulose source such as cotton linter. The onset and end thermal degradation temperatures as well as the maximum weight loss of this cellulose triacetate were reported to be 290–320, 380–400, and 350–360 °C, respectively [29]. Thus, the synthesized cellulose acetate exhibits similar thermal behavior; therefore, such cellulosic resources can be utilized for the derivatization of cellulose.

### 2.6. Morphological Analysis

**Figure 6:**
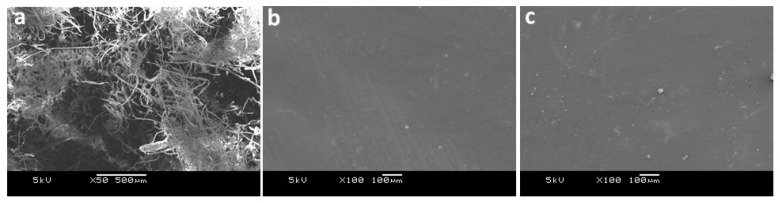
SEM images of (**a**) cellulose, (**b**) CTA-1 and (**c**) CTA-2.

SEM images of cellulose and acetate cellulose are presented in Figure 6. The surface of the cellulose fiber displays defibrillation of the fibrils into individual fibrils of uniform size (Figure 6a). Their smooth and flexible characteristics also indicate that they are free from noncellulosic rigid components such as lignin and other inorganic contaminants such as silica.

These fibers also show increased agglomeration/aggregation due to more surface hydroxyl group exposure, resulting in enhanced intra- and intermolecular hydrogen bonding. This morphology was completely transformed due to the acetylation process, producing a smooth, compact and homogeneous surface of cellulose acetate (Figure 6b,c). However, variations in CTA morphology have been documented, including a spongy nature [13], a porous structure [14], and stacked sheets of thin acetate fibers [30]. These variations can be attributed to differences in the synthetic conditions and operating parameters during analysis.

## 3. Materials and Methods

### 3.1. Materials

The chemicals used in this study were acetic acid (CH_3_COOH, 99%), hydrochloric acid (HCI, 37%), sulfuric acid (H_2_SO_4_, 95–97%) from Sigma Aldrich, USA. Acetic anhydride (C_4_H_6_O_3_, 99%), ethanol (CH_3_CH_2_OH, 96%), acetone (CH_3_COCH_3_), and chloroform (CHCl_3_) were obtained from Panreac Química, Spain. Sodium hydroxide, sodium bisulfate, sodium acetate, and phenolphthalein indicator were purchased from Loba Chemie, India. These reagents were used as received without purification. Commercial cellulose ester (CA-435-75S) was procured from the Eastman Chemical Company, USA. Similarly, pure cellulose was isolated from date palm trunk mesh fibers. The details of the pretreatments and isolation methods are described in our previous work. In brief, fine powder was obtained from palm tree trunk mesh and pretreated with supercritical carbon dioxide (ScCO_2_) to eliminate the water soluble extractives. It was then treated with a 20% (*w*/*v*) sodium hydroxide solution at 90 °C for 6 h. The solid fraction was separated and washed until it was alkali-free. Finally, a bleaching reaction was carried out at 70 °C for 4 h using an acidic solution of sodium chlorite (pH 3.7). The pure cellulose was collected by filtration, washed several times until it becomes neutral, and finally dried to obtain a constant weight. The average yield of the samples was assessed by repeating the experiment in triplicate, resulting in a yield of 65%. Similarly, the alpha and hemicellulose content of this cellulose was determined using the Technical Association of the Pulp and Paper Industry’s (TAPPI) standard method (T 203 cm-09). Moreover, TAPPI standard procedure, T 222 om-98 and T 211 om-07 was utilized to assess lignin and ash content, respectively. This cellulose contains 94% alpha-cellulose, 5% hemicellulose, 0.32% lignin, and 0.23% ash [18].

### 3.2. Preparation of Cellulose Ester

The acetylation of cellulose was conducted using a process described elsewhere [9]. In brief, cellulose was dried in an ordinary vacuum oven at 110 °C for 24 h before use. Ten grams of cellulose was placed in a two-neck round bottom flask equipped with an overhead stirrer. After adding 250 mL of acetic acid, the reaction mixture was maintained at 0–5 °C for one hour. An acetylating mixture comprising 75 mL of acetic anhydride, 0.5 mL of sulfuric acid, and 0.50 g of sodium bisulfate was then added while maintaining this temperature. Then, the flask was allowed to attain a room temperature of 30 °C after one hour. The reaction mixture gradually became viscous after some time, and the reaction continued for another 18 h with stirring.

Finally, to eliminate the sulfate group substituent on the cellulose molecule, a solution of 0.5 g of sodium acetate in 10 mL of glacial acetic acid was added to the reaction mixture one hour before the completion of the reaction. Then, the product was precipitated by pouring into 2 L of distilled water with constant stirring. It was filtered and washed until the product was free from acetic acid odor, and the filtrate attained a neutral pH. The final washing was performed with acetone, and the product was filtered and dried for one day in a vacuum oven at 70 °C. The average yield percentage from three experiments is reported in Table 3. The commercial standard cellulose acetate and synthesized cellulose acetate were termed CTA-1 and CTA-2, respectively.

## 4. Characterization

### 4.1. Acetylation Ratio and Degree of Substitution (DS) Determination

Acetylation ration and degree of substitution were calculated as per ASTM standard method [31]. Briefly, in a 250 mL Erlenmeyer conical flask, 0.5 g of cellulose acetate and 25 mL of 75% aqueous ethanol were added and then heated for a half-hour at 60 °C. Then, 25 mL of 0.49 N NaOH solution was added to each sample and again heated at 60 °C for another half hour. The flasks were closed and left at room temperature with stirring for 72 h. The control samples were also prepared in the same manner. HCl (0.5 N) was used to titrate the excess alkali in the sample and control using phenolphthalein as an indicator. The disappearance of the pink color indicated complete neutralization of the alkali. The slight excess of acid was then back titrated with sodium hydroxide to a phenolphthalein end-point. The degree of substitution value and acetyl percentage were carried out in triplicate and calculated by using the following equations, and the characteristics of CTA are given in Table 3:

(1) $$\%\;Acetyl=\frac{\left[\left(\;NaOH_{iv\;}+NaOH_{lv\;}\right)\times NaOH_{M\;}-HCI_{V}\times HCl_{M}\right]43.10}{W}$$

where

*iv*—initial volume of NaOH
*lv*—last volume of NaOH
*_M_*—molarity of NaOH
*v*—volume of HCl used
*_M_*—molarity of HCl
*W*—weight of sample

(2) $$Degree\;of\;substitution=\frac{3.82\times \%\;Acetyl}{102.4-\%\;Acetyl}$$

### 4.2. Molecular Weight Determination

Gel permeation chromatography (GPC) experiments were carried out at room temperature using a Dionex GPC (Thermo Scientific, Waltham, MA, USA) with a RI detector. To estimate the molecular weights of cellulose acetate samples, a polystyrene calibration curve (10 samples with weight average molecular weights ranging from 164 to 1.5 million) was used. Cellulose acetate (15–20 mg) was dissolved in 5 mL of chloroform, and 20 µL of the solution was injected. The average molecular weight is given in Table 3.

### 4.3. Mechanical Properties

The mechanical properties of the CTA film were estimated by using a Tinius Olsen (Hounsfield H100 KS Series, Salfords, UK) universal testing machine as per ASTM D-882-10. The load cell used was 1 kN with a 5-mm/min crosshead speed. The dog bone type test specimens were made from thick film using a hand press machine (HK 800, Berg and Schmid GmbH, Remseck, Germany) to cut the films with a standard die. The dimensions were 75 mm overall length, 25.0 ± 0.5 mm gauge length, 4 mm width, and 0.2 mm thickness. The test was performed under ambient conditions, and an average value of three measurements is reported in Table 3.

### 4.4. Chemical Functional Group Analysis

Attenuated total reflectance-Fourier transform infrared spectroscopy (ATR-FTIR) analysis was carried out using a Nicolet iN10 FTIR microscope (Thermo Scientific, Winsford, UK) with a Germanium microtip to determine the functional groups of the initial and final products. The analysis was carried out in the wavenumber range of 650–4000 at 4 cm^−1^ over 16 scans.

### 4.5. ^1^H-NMR and ^13^C Analysis

A Jeol resonance 500 MHz NMR (Joel, Tokyo, Japan) spectrometer was used to detect proton nuclear magnetic resonance (^1^H-NMR) and carbon nuclear magnetic resonance (^13^C-NMR) spectra using deuterated chloroform (CDCl_3_) as the solvent. The chemical shifts were reported as parts per million (ppm) in the δ scale downfield from tetramethylsilane (TMS). Signal positions were determined relative to the residual proton signal in CDCl_3_ (δ = 7.3 ppm) and the carbon signal for CDCl_3_ (δ = 77.00 ppm).

### 4.6. Wide-Angle X-ray Diffraction (WAXRD)

The crystalline behavior of the CTA samples was examined using wide-angle X-ray diffraction (WAXRD, D8 Advance, Bruker, Berlin, Germany). A computer-controlled wide-angle goniometer coupled to a sealed-tube source of Cu-Kα radiation (λ = 1.54056 Å) was used. All samples were scanned at 5°/min and 2θ ranged from 5° to 45°.

### 4.7. Thermal Characterization

Thermogravimetric analysis (TGA) was performed using a Shimadzu thermal analyzer (DTG 60H, Shimadzu., Kyoto, Japan). The alumina pan was filled with ~10–15 mg of the sample. Subsequently, the samples were heated from room temperature to 600 °C at a heating rate of 20 °C/min. The analysis was performed under a nitrogen atmosphere with a flow rate of 50 cm^3^/min, and accordingly, the corresponding weight loss was recorded.

### 4.8. Morphological Analysis

Scanning electron microscopy (SEM, JSM-6360A, JEOL, Tokyo, Japan) was used to investigate the surface morphology. The solvent cast thin film (chloroform, ~0.2 mm thick) was placed on conducting carbon tape for analysis. The accelerating voltage was kept at 5 kV, and all of the samples were gold-sputtered before observation to avoid samples being overcharged.

## 5. Conclusions

The significant findings of the present work demonstrated the successful utilization of cellulose from date palm truck mesh to obtain cellulose acetate with properties equivalent to those of commercial derivatives. A commercially viable heterogeneous acetylation procedure was employed, and complete conversion of hydroxyl to acetate groups was achieved by using acetic anhydride as an acetyl donor. The properties of the obtained cellulose triacetate were compared with those of the standard sample and found to have equivalent characteristics. The synthesized cellulose acetate has a degree of hydroxyl substitution (DS) of 2.9 and acetyl percentage of 45, an average molecular weight of 205,102 gmole^−1^, and is completely soluble in chloroform (cellulose acetate with a lower DS is soluble mainly in acetone and/or water). In addition, Fourier transform infrared spectroscopy and ^1^H and ^13^C nuclear magnetic resonance spectroscopy revealed the complete structural elucidation of cellulose acetate. The XRD analysis indicated the semicrystalline nature of the sample, while thermal analysis showed good thermal stability compared to the standard samples. From morphology examination, the resulting cellulose acetate showed a smooth, uniform, and compact surface. Nonetheless, the overall results suggest that the cellulose ester prepared in this work can be utilize as a polymer matrix for cellulose-based biodegradable polysaccharides composite applications.

## Figures and Tables

**Table 1:** Characteristic peak assignment of cellulose and cellulose triacetate. Adapted with permission from Ref. [23,24]. Copyright 2002 and 2004 Elsevier. Ref. [25]. Copyright 2015 American Chemical Society.

Functional Group Assignments	Wavenumber, cm^−1^		
	Cellulose	CTA-1	CTA-2
ν_OH (_covalent bond, hydrogen bonding)	3335	--	--
ν_CH_	2897	2940	2952
C=O stretching of the acetyl group	--	1740	1732
H_2_O absorbed (absorbed water hydrogen-bonded))	1645	1635	1640
δ_CH2_ ((symmetric) at C-6; crystalline region)	1430	1430	1425
C–H bending vibration of CH_3_ in the acetyl group)	--	1369	1365
δCH_2_ (wagging at C-6) Or δ_COH_ in a plane at C-2 and C-3	1320	1319	1322
δCOH in a plane at C-6	1253	--	--
C–O stretching of the acetyl group	--	1222	1215
ν_CO_, δ_OH_ (γCOC at β-glycosidic linkage)	1160	1168	1164
γ-ring in plane	1103	1122	1125
ν_C-O_ (c-o-c of the cellulose backbone)	1052	1037	1040
γ-CO at C-6	1029	952	1010
δ_CH2_ (γCOC at β-glycosidic linkage; amorphous region)	898	899	902

**Table 2:** Carbon signal positions of cellulose acetate samples. Reprinted/Adapted with permission from Ref. [14]. Copyright 2014 John Wiley and Sons. Ref. [26]. Copyright 2013 Elsevier.

Sample	C7 = O (ppm)	C1 (ppm)	C4 (ppm)	C3 (ppm)	C5 (ppm)	C2 (ppm)	C6 (ppm)	C8 (ppm)
CTA-1	170.3 (C_6_) *	100.6	76.2	71.9	72.9	71.9	62.1	20.9 (C_6_) ^$^
	169.8 (C_3_) *							20.6 (C_3_) ^$^
	169.4 (C_2_) *							20.5 (C_2_) ^$^
CTA-2	170.3 (C_6_) *	100.6	76.2	71.9	72.9	71.9	62.2	20.9 (C_6_) ^$^
	169.9 (C_3_) *							20.7 (C_3_) ^$^
	169.4 (C_2_) *							20.6 (C_2_) ^$^

* C7 is bonded to C6, C3, and C2.; ^$^ C2, C3 and C6 bonded to C8.

**Table 3:** Characteristics of CTA samples.

Sample	%Acetyl	DS	%Yield	Average Mol. Wt. (Mw)	Tensile Strength (MPa)
CTA-1	44.44 (±1.98)	2.91 (±0.032)	-	222,168.60	27 (±2.65)
CTA-2	43.51 (±1.15)	2.85 (±0.046)	94.5 (±2.68)	205,102.25	22 (±3.00)

## Data Availability

Data are contained within the article.

## Sample Availability

Samples of the compounds are available from the corresponding author upon request.
